# Multiclass Classification for the Differential Diagnosis on the ADHD Subtypes Using Recursive Feature Elimination and Hierarchical Extreme Learning Machine: Structural MRI Study

**DOI:** 10.1371/journal.pone.0160697

**Published:** 2016-08-08

**Authors:** Muhammad Naveed Iqbal Qureshi, Beomjun Min, Hang Joon Jo, Boreom Lee

**Affiliations:** 1 Department of Biomedical Science and Engineering (BMSE), Institute of Integrated Technology (IIT), Gwangju Institute of Science and Technology (GIST), Gwangju, Republic of Korea; 2 Department of Neurologic Surgery, Mayo Clinic, Rochester, Minnesota, United States of America; Tianjin University, CHINA

## Abstract

The classification of neuroimaging data for the diagnosis of certain brain diseases is one of the main research goals of the neuroscience and clinical communities. In this study, we performed multiclass classification using a hierarchical extreme learning machine (H-ELM) classifier. We compared the performance of this classifier with that of a support vector machine (SVM) and basic extreme learning machine (ELM) for cortical MRI data from attention deficit/hyperactivity disorder (ADHD) patients. We used 159 structural MRI images of children from the publicly available ADHD-200 MRI dataset. The data consisted of three types, namely, typically developing (TDC), ADHD-inattentive (ADHD-I), and ADHD-combined (ADHD-C). We carried out feature selection by using standard SVM-based recursive feature elimination (RFE-SVM) that enabled us to achieve good classification accuracy (60.78%). In this study, we found the RFE-SVM feature selection approach in combination with H-ELM to effectively enable the acquisition of high multiclass classification accuracy rates for structural neuroimaging data. In addition, we found that the most important features for classification were the surface area of the superior frontal lobe, and the cortical thickness, volume, and mean surface area of the whole cortex.

## Introduction

The neurodevelopmental disorder of attention deficit hyperactivity disorder (ADHD) has a prevalence of 3.4% among children and adolescents [1]. Although the statistic itself seems to have decreased from 5.3% as reported by [2], the burden ADHD places on patients, their families, and the societies to which they belong remains significant. Usually, the disorder begins to influence patients from an early age and affects their entire life, and without appropriate treatments, the illness leads to a poor prognosis. Thus, the diagnosis of ADHD is important. Children affected by the disorder have characteristic symptoms such as attention deficit, hyperactivity, and impulsiveness. Currently, it is believed that this characteristic manifestation of symptoms originates from the dysfunction of related cognitive processes [3]. In addition, the underlying mechanisms of ADHD seem to be associated with delayed cortical development [4]. On the other hand, according to the Diagnostic and Statistical Manual of Mental Disorders, fifth edition (DSM-5), there are three types of ADHD, based on the predominant symptoms: 1) predominantly inattentive presentation, 2) predominantly hyperactive-impulsive presentation, and 3) combined presentation [5]. In addition, the results of many studies that have investigated these subtypes of ADHD such as [6–8] have been published. However, the outcomes of a small number of studies that attempted to distinguish among the features of these subtypes with neuroimaging methods were inconclusive [9, 10].

In recent years, many studies based on classification using neuroimaging data for discriminating the clinical diagnosis have been published. In fact, the clinical experience of the psychiatrist, a detailed record of the history of the patient, and other information resulting from interviews are important. However, the past decade has seen an increase in interest in the use of machine-learning techniques, not only in the field of psychiatry, but also in other medical fields such as protein folds and bioinformatics data prediction and classification [11–13]. Previously, a number of studies using neuroimaging methods in the psychiatric research field have been reported [14–16]. Consequently, with the familiarity and understanding of neuroimaging methods, a series of candidate features such as cortical thickness, volumetric data, demographic information, and other fMRI data have been used for machine learning in studies related to this field. This kind of machine-learning-based approach serves as a very powerful tool for neuroimaging studies and plays an important role in terms of the integration of machine learning and neuroscience. The comparatively higher resolution of structural MRI data makes these data a more appropriate choice for use in classification experiments. The most abundantly used machine-learning tool in the neuroimaging community is SVM. Our work involves a comparative study by using a recently proposed H-ELM [17] classification framework in comparison to SVM and basic ELM, of which H-ELM achieved the highest accuracies both for binary and multiclass settings.

As previously reported in several studies, certain numbers of anatomical regions were identified as being affected by ADHD. A recent study by [18] reported the abnormal surface morphology of the central sulcus in children with ADHD; specifically, they found that the average depth and maximum depth of the left central sulcus, as well as the average cortical thickness value of the bilateral central sulcus in the ADHD group, were significantly larger than those in healthy children. ADHD is responsible for a reduction in the surface area and cortical volume up to 7% bilaterally and 7-8%, respectively [19]. In the anterior brain region, posterior brain regions including the left and right superior temporal and parietal lobes, temporal junction, and insula, evident anatomical abnormalities can be observed from the cortical thickness and folding analysis [20, 21]. The studies by [22] showed a total reduction of 3–5% in cerebral volume. Abnormalities in several specific regions including the callosum, striatum, cerebellum, lateral prefrontal cortex, and cingulate cortex were reported by [22]. Thus, these regions can be used as potential biomarkers for the automatic diagnosis of ADHD by using machine-learning approaches.

Our work focused on the differential diagnosis of the subtypes of ADHD based on machine learning. Currently, one of the reasons for the increasing tendency to implement machine learning for the diagnosis of psychiatric disease is that the diagnostic process mainly depends on what is determined by experienced psychiatrists. The present study utilized the implementation of an H-ELM classifier to distinguish ADHD-combined-type and ADHD-inattentive-type patients from typically developing children. Additionally, to improve the classification accuracy, we utilized the standard RFE-SVM algorithm proposed by [23] and available at http://people.kyb.tuebingen.mpg.de/spider/help_rfe.html. This algorithm accepts five different types of cortical feature data as input. The cortical feature measures include mean cortical thickness, surface area, folding indices, mean curvature indices, and the volume of the predefined “Desikan Killiany Tourville” (DKT) atlas-based ROI [24] segments. In addition, we performed an ANOVA test based on group analysis to identify the significantly different anatomical regions among the three groups namely, ADHD-I, ADHD-C, and TDC.

The remainder of this paper is organized as follows. Section 2 provides details of the dataset, subject selection, preprocessing of the MRI data, an introduction of the classification algorithms, feature selection and optimization, permutation testing for validation of the results, and statistical analysis. Section 3 presents the comparative results of both the multiclass and binary classifiers by using H-ELM, ELM, and SVM. In addition, this section also includes the ANOVA test results. Section 4 includes the discussion and conclusion of the article.

## Materials and Methods

### MRI Dataset

We obtained neuroimaging data of ADHD patients from the ADHD-200 MRI dataset, which is publicly available at http://fcon_1000.projects.nitrc.org/indi/adhd200. These T-1-weighted MRI scans were acquired at six different institutes with participant ages ranging from 7 to 14 years. The sites were Brown University (BU), New York University Child Study Center (NYU), Beijing Normal University (BNU), the Kennedy Krieger Institute (KKI), Oregon Health and Science University (OHSU), and Washington University in St. Louis (WU). All participants were scanned using 3.0-Tesla scanners. The other technical details about the scanner parameters from each participating site are already available at the above-mentioned URL of the ADHD-200 global competition. In addition, all the sites contributing to ADHD-200 had approval from their local institutional review board (IRB) and complied with local IRB protocols.

### Subjects

The ADHD-200 dataset included more than 1000 subjects; however, we followed a balanced design approach and only chose 53 subjects from each of the three groups: typically developing children (TDC), ADHD inattentive type (ADHD-I), and ADHD combined type (ADHD-C). The main focus of the current study was to elaborate the significance of a supervised multiclass classification between TDC, ADHD-C, and ADHD-I based on the H-ELM framework and all the subjects were chosen from the training part of the ADHD-200 dataset, which consists of 776 subjects. Each group contained 9 female and 44 male subjects. There may have existed some intrinsic bias in the data regarding batch effects as well as hardware bias due to multisite data collection [25]. However, we retained the bias as default for the criteria for selecting subjects for this study. The IQ information of a few subjects was not provided by some of the collection sites; therefore, we did not use this information in the present study. More detailed demographic information can be acquired from the same resources listed above. Information regarding our selected subjects is summarized in Table 1.

**Table 1:** Demographic variables of the participants.

Groups	TDC	ADHD-I	ADHD-C
Number of subjects	53	53	53
Age (*mean* ± *SD*)	12.75 ± 3.86	12.42 ± 2.23	11.83 ± 3.52
Full IQ (*mean* ± *SD*)	114.86 ± 13.86	102.47 ± 13.11	110.10 ± 13.88
Handedness	Right only	Right only	Right only

Abbreviation: TDC = typically developing children; ADHD-I = attention-deficit/hyperactivity disorder, inattentive type; ADHD-C = attention-deficit/hyperactivity disorder, combined type; SD = standard deviation

### Preprocessing of Structural MRI Data

The raw data on their own provide very little information; therefore, preprocessing is required to extract the patterns of useful knowledge [26]. All of the structural MRI images were preprocessed using the fully automated pipeline of FreeSurfer 5.3.0 [27–31] for volumetric segmentation and cortical reconstruction. The software proceeds with motion correction, T1-weighted image averaging, and registration to the Talairach space, followed by skull stripping with a deformable template model. The white and pial surface was generated for each hemisphere. A cortical surface-based atlas (DKT atlas) was mapped to a sphere aligning the cortical folding patterns, which provided accurate matching of the morphologically homologous cortical locations across subjects. For each of the DKT31 protocol-based segments, Freesurfer calculated nine different measures, including, surface vertices, surface area, gray matter volume, average cortical thickness, cortical thickness standard deviation, cortical mean curvature, cortical Gaussian curvature, cortical folding index, and cortical curvature indices [25]. We used five of the above-mentioned measures in this study. The surface area was calculated by computing the area of every triangle in a standardized spherical surface tessellation. The average shortest distance between white and pial surfaces denotes the cortical thickness at each vertex of the cortex. The local curvature was computed using the registration surface based on the folding patterns. The folding index over the whole cortical surface was measured for each subject [19].

After preprocessing, the subcortical regions were masked to separate the significant cortical data by using AFNI. Finally, the cortical thickness data were converted into surface maps using the AFNI program MapIcosahedron. These maps were then subjected to heat-kernel-based smoothing using the AFNI program SurfSmooth with a 30-mm kernel.

### ANOVA for Cortical Thickness

First, we performed an ANOVA to examine and visualize the differences of cortical thickness among the three groups. The AFNI program 3dANOVA2 [32] was used for the analysis. A multiple comparison test was performed using the AFNI program 3drefit with the FDR correction. In addition, the AFNI program SurfClust was used to find regions that have significant differences between groups with an area of more than 50*mm*^2^. The *p*-value threshold was set to be 0.001.

### Classification

In this study, three machine-learning classification algorithms were used namely, H-ELM, ELM, and SVM. After comparing the results of all the classifiers, H-ELM proved to be the most efficient algorithm both in terms of computation time and accuracy. A brief description of each method is provided in the following paragraphs.

#### Hierarchical-Extreme Learning Machines

The extreme learning machine, which was originally proposed by [33], is a comparatively newer machine-learning algorithm. It was adopted by many previous neuroimaging studies [19, 34, 35] as the main tool for data discrimination in the binary setting. However, to the best of our knowledge, the present study is the first in this domain that utilized the recently proposed H-ELM framework for the multiclass classification of neuroimaging data.

The H-ELM framework consists of two main parts, an ELM-based unsupervised sparse multilayer autoencoder for feature encoding and supervised ELM for classification. The ELM is an effective solution for single-layer feed-forward networks (SLFNs). Unlike SVM and other back-propagation methods, the parameters of the hidden layer need not be tuned and can be generated randomly before the training samples are acquired. Unlike conventional ELM, which is used for comparison purposes in this study, H-ELM is a multi-layer learning architecture and uses an ELM-based sparse autoencoder for feature learning via *ℓ*_1_−norm optimization to generate more sparse and meaningful hidden layers of features before classification by the original ELM.

Mathematically, in the first step, the output of each hidden layer can be represented as

$$\begin{array}{c} H_{j}=\lambda \left(H_{j-1}\cdot \beta \right), \end{array}$$ (1)

where **H**_*j*_ is the output of the *j*th layer (*j* ∈ [1,*K*]), **H**_*j*−1_ is the output of the (*j*−1)^*th*^ layer, λ(⋅) denotes the activation function of the hidden layers and ***β*** represents the output weights.

In the second step, an original SLFN ELM with *m* hidden nodes is used for classification. It can be represented as

$$\begin{array}{c} f_{m}\left(\rho \right)=\sum_{j=1}^{m}\eta_{j}\left(\rho ,\theta_{j},\gamma_{j}\right)\cdot \beta_{j},\;\;\theta_{j}\in R^{d},\gamma_{j},\beta_{j}\in R^{1} \end{array}$$ (2)

where *η*_*j*_(⋅) denotes the *j*^*th*^ hidden node activation function, ***θ***_*j*_ is the input weight vector connecting the input layer to the *j*^*th*^ hidden layer, *γ*_*j*_ is the bias weight of the *j*^*th*^ hidden layer, and ***β***_*j*_ is the output weight. **R**^*d*^ is the d-dimensional and **R**^1^ is the one-dimensional Euclidean space. For the additive nodes with activation function λ, *η*_*j*_ can be defined as

$$\begin{array}{c} \eta_{j}\left(\rho ,\theta_{j},\gamma_{j}\right)=\lambda \left(\theta_{j}\cdot \rho +\gamma_{j}\right) \end{array}$$ (3)

The dot in the Eq (3) denotes the inner product of ***θ***_*j*_ and ***ρ***. Eqs (2) and (3) can be compactly written as

$$\begin{array}{c} B=H^{†}T, \end{array}$$ (4)

where **B** = [***β***_1_, …, ***β***_*m*_]^*T*^ is the weight matrix for *m* hidden nodes, **H** is the hidden layer output matrix of each of the *n* arbitrary distinct training data samples,

$$\begin{array}{c} H=[\begin{array}{ccc} \begin{array}{c} \lambda (\theta_{1}\cdot \rho_{1}+\gamma_{1}) \end{array} & \cdots & \begin{array}{c} \lambda (\theta_{m}\cdot \rho_{1}+\gamma_{m}) \end{array}\\ \vdots & \ddots & \vdots \\ \begin{array}{c} \lambda (\theta_{1}\cdot \rho_{n}+\gamma_{1}) \end{array} & \cdots & \begin{array}{c} \lambda (\theta_{m}\cdot \rho_{n}+\gamma_{m}) \end{array} \end{array}] \end{array}$$ (5)

**H**^†^ = (**H**^*T*^ **H**)^−1^ **H**^*T*^ is the Moore-Penrose inverse of **H**, and $T={\left[t_{1}^{T},\cdots ,t_{m}^{T}\right]}^{T}$ is the target matrix of training data where $t_{1}^{T}=\left[t_{11},\cdots ,t_{1n}\right]$ [17].

The H-ELM framework has a certain number of hyperparameters that need to be tuned to achieve the maximum performance of the classifier in terms of accuracy. In this study, we used the MATLAB implementation of the H-ELM as proposed by [17]. The hyperparameters were the number of nodes in all three layers *N*1, *N*2, *and N*3, *C* was the *ℓ*_2_ penalty of the 3rd layer of ELM, and *s* was the scaling (regularization) parameters. Since there is no proper guideline available for the adjustment of these parameters, we have used a greedy search method to tune these parameters for achieving the maximum validation accuracy before training the classifier. In this study, the search scale for selecting these parameters was set to *N*1 = *N*2 = 48, *N*3 = [49, 50, …, 500], *C* = [2^−31^,2^−32^,…,2^−40^], *and s* = [0.1, 0.2, …1.0]. In addition, the data labels were encoded as *class*1 = [1 −1 −1], *class*2 = [−1 1 −1], and *class*3 = [−1 −1 1]. All the features were normalized and scaled to values between −1 and 1 to improve the performance of the H-ELM framework.

#### ELM Classifier

We used the basic ELM classifier [33] with a sigmoid activation function in this study. The number of nodes was selected by a greedy search method. The search scale was set to *N* = [1, 2, …..,50].

#### SVM Classifier

SVM, which was originally proposed by [36], is useful for classification in the neuroimaging field and has been one of the most popular machine-learning tools in the neuroscience domain in the last decade. It is a supervised classification algorithm, and, intrinsically, it is a binary classification algorithm. We have used this algorithm in both binary and multiclass (more commonly known as one-versus-all, or OVA) settings. It maps features in higher dimensional space using linear and nonlinear functions known as kernels. In this study we used both linear and nonlinear radial basis function (RBF) kernels. The SVM classifier discriminates between the two classes in the feature space by constructing a hyperplane that discriminates the training data from the maximum margin between the two data clusters. In a multiclass setting, an SVM algorithm was used to classify each individual into one of the three diagnostic groups. The multiclass classification was conducted by building a *K* OVA classification technique. The OVA is an alternative method of applying the SVM in the case of *K* > 2 classes. It functions by comparing one of the *K* classes to the remaining *K*−1 classes. The OVA chooses the class that is selected by the largest number of classifiers as it amounts to a high level of confidence that the test class belongs to the *K*^*th*^ class rather than to any of the other classes.

### Feature Selection and Optimization

In most studies involving neuroimaging analysis, the number of features per subject greatly outnumbers the number of subjects. This concept is commonly referred to as the *curse of dimensionality* [37]. Feature selection can generally be subdivided into two categories: the filter model and the wrapper model. Optimal feature selection provides a subset of features that leads towards optimal classification accuracy.

Usually, an *optimal* feature has relevance to the class concept and represents no overlap with any other feature in the feature set and/or subset. We used a very straightforward filter model for feature measure selection in this study [19, 38]. The FreeSurfer pipeline was used to automatically generate the cortical features of five feature measure types as indicated in Table 2.

**Table 2:** Feature Measures and Cortical Feature Index Information.

Feature Measure (*fM*)	Feature Measure Type	Indices of Cortical Features
*fM*1	Mean Cortical thickness	1–64
*fM*2	Surface Area	65–128
*fM*3	Folding Indices	129–192
*fM*4	Mean Curvature Indices	193- 256
*fM*5	Volume	257–320

The “feature measures (*fM*)” were utilized to acquire 64 cortical features of each type. These 64 cortical features were obtained as a pair of 32 features from each hemisphere. The 32 cortical features consisted of the values of 31 ROIs of DKT atlas-based brain segments and the remaining cortical feature was generated by obtaining the average value of all 31 ROIs of the corresponding hemisphere. Mathematically, this is expressed as follows:

$$\begin{array}{c} Mean\;Value\;\left(MV\right)=\frac{1}{31}\sum_{i=1}^{31}\left(fM\;value\;in\;ROI_{i}\right) \end{array}$$ (6)

$$\begin{array}{c} 5fM\times (31\;ROI+MV)\times 2\;Hemispheres=320\;CorticalFeatures \end{array}$$ (7)

**Fig 1:**
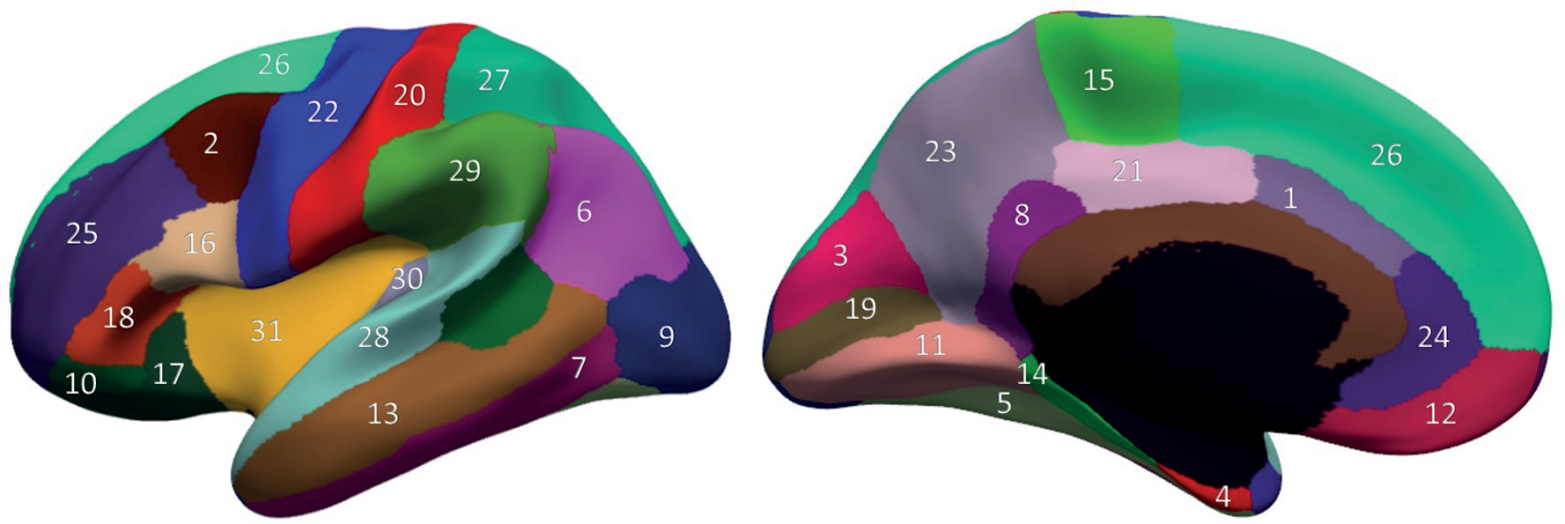
Cortical segmentation based on the DKT atlas. Each of the 31 anatomical ROIs are shown with a specific color and index (see Table 3 for indices). The figures on the left and right show the lateral and medial views, respectively.

The segmentation of cortical ROIs was based on the comparatively new DKT atlas [24]. This atlas is provided as a built-in tool in the Freesurfer software package [28]. These cortical ROIs are shown in Fig 1 with the indices referring to those listed in Table 3.

**Table 3:** DKT31 Protocol-based Cortical ROIs.

No.	Label	No.	Label
1	Caudal Anterior Cingulate	17	Pars orbitalis (IFG)
2	Caudal middle frontal gyrus	18	Pars triangularis (IFG)
3	Cuneus	19	Pericalcarine cortex
4	Entorhinal cortex	20	Postcentral gyrus
5	Fusiform gyrus	21	Posterior cingulate
6	Inferior parietal lobule	22	Precentral gyrus
7	Inferior temporal gyrus	23	Precuneus
8	Isthmus cingulate	24	Rostral anterior cingulate
9	Lateral occipital cortex	25	Rostral middle frontal
10	Lateral orbitofrontal gyrus	26	Superior frontal
11	Lingual gyrus	27	Superior parietal lobule
12	Medial orbitofrontal gyrus	28	Superior temporal gyrus
13	Middle temporal gyrus	29	Supramarginal gyrus
14	Parahippocampal gyrus	30	Transverse temporal gyrus
15	Paracentral gyrus	31	Insula
16	Pars opercularis (IFG)	32	Mean measure

Abbreviation: IFG = Inferior frontal gyrus

The use of a wrapper to achieve model-based feature selection usually consists of two steps: 1) feature subset selection, using the accuracy of the base classifier and 2) using the best feature subset for learning and testing. The wrapper approach consists of using the prediction performance of a base classifier. It performs selection by taking into account the classifier as a black box and ranking the subset of features by their predictive power [26]. The preselected 320 cortical features categorized in Table 2 are subsequently fed into the wrapper model (RFE-SVM) to generate the feature-ranking list in the order of significance.

#### RFE-SVM

**Fig 2:**
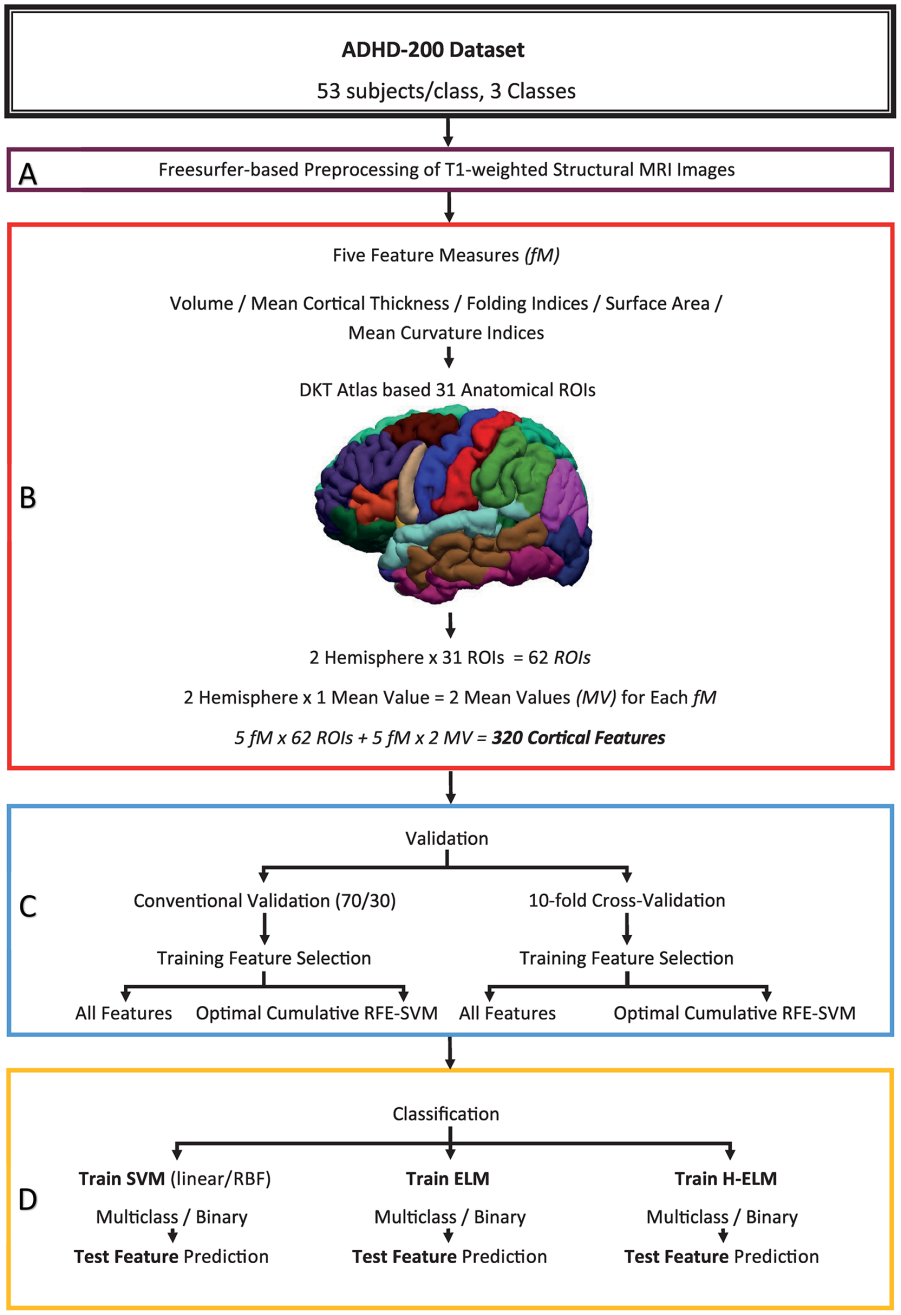
Overall framework of the study. The level-A block represents subject selection from the ADHD-200 dataset and briefly describes the preprocessing step, the level-B block provides information about the 31 DKT atlas-based ROIs, selection of the five feature measures and the total count of the cortical features. The level-C block elaborates the data validation methodologies and feature selection for the classifiers. Level-D represents the classifier choice for both the multiclass and binary settings.

We improved the classification accuracy by applying another fold of feature selection by using RFE-SVM on the preselected cortical features provided in Table 2. This is a wrapper-based method and selects feature subsets by utilizing greedy backward selection. A linear SVM classifier serves as the base algorithm and removes the features with the smallest absolute value it returns in each cycle of the training iteration. RFE-SVM continues the iteration until it reaches the specified feature count in the base algorithm call. During this training process, RFE-SVM generates a feature-ranking list. RFE-SVM uses the accuracy of cross-validation to generate the feature subset, thereby possibly avoiding the over-fitting problem as well [22]. This method was successfully deployed in many previous research efforts related to ADHD-200 dataset classification, including [22], and a detailed motivation for choosing this method for feature selection can be found in [25]. Fig 2 depicts the overall framework of the current study.

### Permutation Testing

Permutation testing is used to assess the statistical significance of the classifier performance [39]. Briefly, the assessment proceeds as follows. First, we choose the test statistic of the classifier, permute the class labels for the training dataset randomly and assign them to the classifier, and check for cross-validation. If the classifier can learn the relationship between the data and their labels with a p-value exceeding 0.05, the test result is considered as insignificant and the null hypothesis is considered to be true. Generally, a lower p-value of the permuted prediction rate against the prediction rate with the original data labels indicates a higher significance of the classifier performance. As there is no fixed rule for setting the number of permutations, in the current study, we permuted the data 10,000 times.

### Validation, Training, and Testing Subjects

We used two methods of validation, i.e., conventional 70/30 partitioning, and k-fold (k = 10) cross-validation. In the case of conventional partitioning, we divided the data into training and testing datasets and used the first 36 randomly chosen subjects from each group for training and the remaining 17 subjects for testing the classifier. In the 10-fold cross-validation, all 159 subjects were randomly divided into 10 equally sized subsets, i.e., testing (10%) and training (90%) subjects for each of the 10 folds of the cross-validation. In addition, for nested validation, we repeated the classification experiment 10 times in the case of 10-fold cross-validation and 100 times in the case of conventional validation to ensure the robustness of the classification results. The mean accuracy of all the repetitions was reported as the final result.

### Performance Evaluation Methods

The performance of the classifier was measured in terms of classification accuracy and the Kappa score for multiclass classification. In addition, we calculated the confusion matrix for each iteration cycle of the classifier. In the binary case, besides accuracy, there exist many measures, including the area under the receiver operating characteristic (ROC) curve (AUC), sensitivity, specificity, F-score, and J-Statistic. **Classification accuracy** can be measured as

$$\begin{array}{c} Accuracy=\frac{\left(TP+TN\right)}{\left(TP+TN+FP+FN\right)}*100, \end{array}$$ (8)

where *TP* is true positive, *TN* is true negative, *FP* is false positive, and *FN* is false negative. These values were obtained from the confusion matrix. **Kappa score** is considered as a robust statistical measure for the assessment of multiclass classifiers. It is defined by [40] as

$$\begin{array}{c} k=\frac{Pr(a)-Pr(e)}{1-Pr(e)}, \end{array}$$ (9)

where *Pr*(*a*) is the relative observer agreement between raters and *Pr*(*e*) is the hypothetical probability of chance agreement. The value of *k* ranges between +1 and −1, where *k* = 1 represents perfect classification, *k* = 0 represents chance level, and *k* = −1 represents completely erroneous classification [41].

## Results

### Multiclass Classification

The OVA class setting was used to perform the multiclass classification. We performed the classification on both the complete and reduced cortical features for the multiclass (3 classes) classification and applied an RFE-SVM feature selector on the cortical data to achieve a high classification score. As a result, after 10-fold cross-validation, the average overall accuracy was 57.81%, whereas the use of conventional validation enabled us to achieve 60.78% accuracy with H-ELM. Details of the classification scores (accuracy and Kappa) using all of H-ELM, ELM, and SVM (with RBF and linear kernels) are presented in Table 4. We also performed a similar classification experiment with 10-fold cross-validation and conventional validation on the complete dataset with all the features; overall, the average maximum accuracy for the latter part with 10-fold cross validation was 51.59%, whereas the use of conventional validation achieved 56.87% accuracy with H-ELM. Hence, in view of the results presented in Table 4 we can suggest that, by using H-ELM, the accuracy of the testing classification between multiple diagnosis groups can be increased.

**Table 4:** Mean Multiclass (3 classes) classification results.

**RFE-SVM feature selection**				
*Classifier*	H-ELM	ELM	SVM linear	SVM RBF
10−*fold CV* (%)	57.81 (*p* < 0.0001)	54.02	49.06	49.69
*Kappa score*	0.3675	0.3118	0.2358	0.2453
*Conventional Validation* (%)	60.78 (*p* < 0.0001)	54.90	43.14	45.10
*Kappa score*	0.4118	0.3235	0.1471	0.1769
**All features**				
*Classifier*	H-ELM	ELM	SVM linear	SVM RBF
10−*fold CV* (%)	51.59 (*p* < 0.0001)	40.75	43.40	42.77
*Kappa score*	0.2747	0.1101	0.1509	0.1415
*Conventional Validation* (%)	56.87 (*p* < 0.0001)	39.22	31.37	39.22
*Kappa score*	0.3529	0.0882	-0.0294	0.0882

Abbreviations: H-ELM = hierarchical extreme learning machine; ELM = extreme learning machine; SVM = support vector machine; RBF = radial basis function; RFE = recursive feature elimination; CV = cross-validation

### Binary Classification

Although the main focus of the present study is to prove the significance of multiclass classification results, we have also shown binary classification results to justify the multiclass classification results. In general, the multiclass classification should preferably show at most equal and ideally lower overall accuracy than any of the binary combinations of data groups. Our binary classification results help us to demonstrate this fact.

Three binary classifications were performed on the preprocessed cortical dataset by using all of the H-ELM, ELM, and SVM (with RBF and linear kernel) classifiers. We applied the RFE-SVM-based significant feature selector on the cortical data to acquire the optimal features. We achieved maximum accuracy of 85.29%, 79.40%, and 73.58% in the case of H-ELM, ELM, and SVM, respectively. Details of the classification score (accuracy) for each disease group are presented in Table 5.

**Table 5:** Binary classification results.

**RFE-SVM feature selection, 10-fold Cross-Validation**			
*Classifier*	ADHD-C-ADHD-I	ADHD-C-TDC	ADHD-I-TDC
*H*−*ELM* (%)	80.30 (*p* < 0.0001)	77.64 (*p* < 0.0001)	80.30 (*p* < 0.0001)
*ELM* (%)	74.24	74.73	74.58
*SVMlinear* (%)	73.58	71.70	68.86
*SVMRBF* (%)	66.98	64.15	57.54
**RFE-SVM feature selection, Conventional Validation**			
*Classifier*	ADHD-C-ADHD-I	ADHD-C-TDC	ADHD-I-TDC
*H*−*ELM* (%)	79.41 (*p* < 0.0001)	79.40 (*p* < 0.0001)	85.29 (*p* < 0.0001)
*ELM* (%)	76.47	79.40	79.40
*SVMlinear* (%)	73.52	64.71	70.59
*SVMRBF* (%)	58.82	64.71	61.76

Abbreviation: H-ELM = hierarchical extreme learning machine; ELM = extreme learning machine; TDC = typically developing children; ADHD-I = attention-deficit/hyperactivity disorder-inattentive type; ADHD-C = attention-deficit/hyperactivity disorder combined type; SVM = support vector machine; RBF = radial basis function; RFE = recursive feature elimination

### Classification Performance on Selected Features

The RFE-SVM feature selection method was used to arrange the 320 cortical features of the data in descending order according to the feature rank indicated in each cycle of the iteration. We combined each feature with all preceding feature rows as a ranked-classification dataset (*RCD*) for each iteration cycle. Even though the feature ranking changes in each cycle of the iteration, all the features selected in the previous iteration cycle stay in the selected subgroup but with a different rank for each cycle according to their significance level. Next, conventional 70/30 and 10-fold cross-validation was applied to compare the performance of all of the H-ELM, ELM, and SVM classifiers in ADHD classification. The first 46 *RCDs* include all the peak classification rates in the binary settings. In the case of a multiclass setting, *RCD*_126_ has the highest accuracy with 10-fold cross-validation.

**Fig 3:**
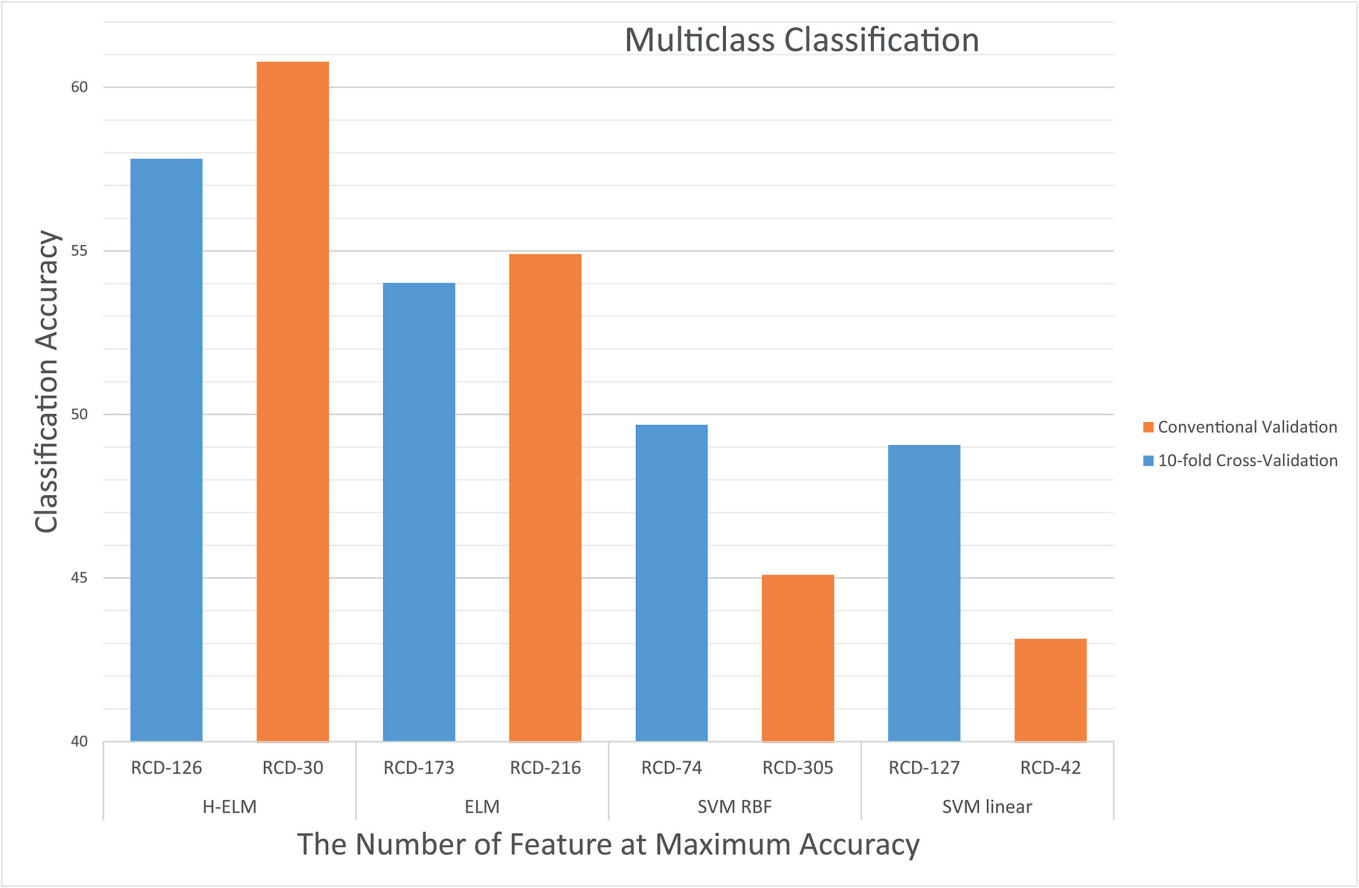
Comparison of the testing accuracy of H-ELM, ELM, SVM linear, and SVM RBF in a multiclass setting using conventional validation and 10-fold cross-validation with RFE-SVM. *RCD* represents the ranked classification dataset size acquired by cumulative RFE-SVM to achieve the highest accuracy.

Fig 3 shows a comparison of the multiclass classification rates of all four of the H-ELM, ELM, SVM linear, and SVM-RBF classifiers used in this study. The classification rates are shown for each of the experimental datasets until we achieve the highest accuracy at the corresponding *RCD*.

The permutation testing was performed with 10,000 iterations using the top *RCD* features. The maximum accuracy rate with the corresponding type of validation was used as the test statistic in both binary and multiclass classifications. The test reflected that the H-ELM classifier learned the relationship between the data and the labels with high significance of p < 0.0001 in both binary and multiclass classifications.

### ANOVA Analysis for Cortical Thickness

**Fig 4:**
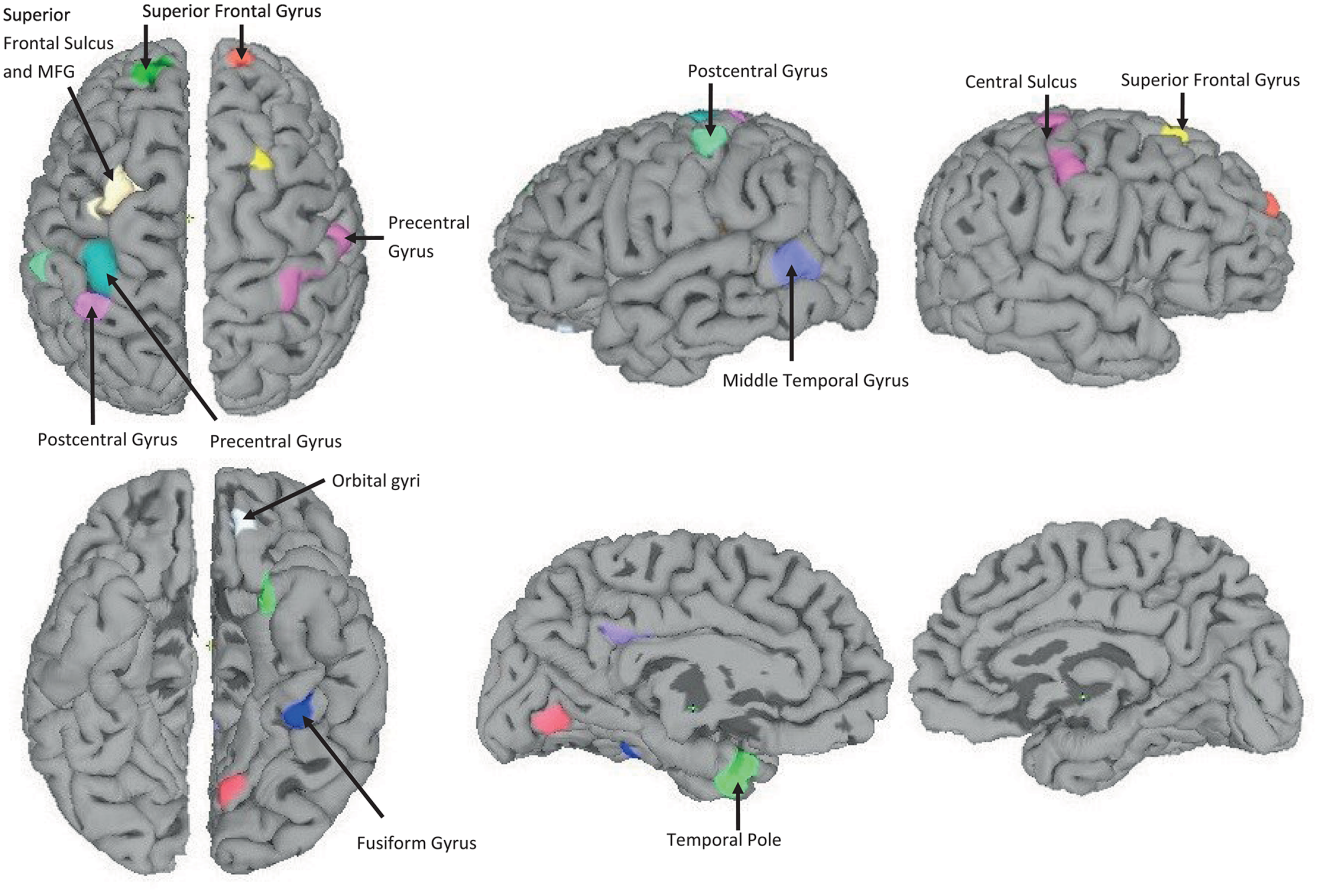
Twelve regions with significant differences as determined by ANOVA. The left column shows the transverse view. The middle and right figures in the first row show the lateral view. The middle and right figures of the second row show the medial view. The most significant results are located in the superior frontal gyrus.

ANOVA tests showed 10 areas of significant thickness change in the left hemisphere and 2 areas in the right hemisphere (*p* < 0.001, cluster size 50*mm*^2^) (see Fig 4). In the post-hoc test, we found that the TDC group had 8 areas (left: middle frontal gyrus, precentral gyrus, dorsal and middle postcentral gyrus, middle temporal gyrus, temporal pole, intraparietal sulcus; right: precentral gyrus and central sulcus) that had the highest thickness value as compared to other groups. In contrast, the ADHD-I group had 3 areas (left: orbital gyri and fusiform gyrus; right: superior frontal gyrus) with the highest thickness value. However, none of the highest values was found in any region within the ADHD-C group.

Our study also confirms the previous findings of anatomical abnormalities as mentioned in the introduction of this paper. These regions can be used as potential biomarkers for the automatic diagnosis of ADHD by machine-learning approaches. We identified the surface area of the superior frontal lobe (ROI-26) and mean surface area of the whole cortex as being the major contributors to differential diagnosis of ADHD along with the cortical thickness and volume of the whole cortex by using multiclass classification.

## Discussion

The main result of this study was to achieve high multiclass accuracy by using the H-ELM framework on RFE-SVM-based selected features. To the best of our knowledge, this is the first study in which the H-ELM framework was used for multiclass classification on structural MRI data from the ADHD-200 competition dataset. Our results suggest that we can achieve high classification accuracy between the patient and control groups. In every test setting, the H-ELM framework outperformed basic ELM and two SVM classifiers.

In our study, we utilized the SVM-based recursive feature elimination algorithm. This algorithm selects features that represent higher degrees of significance based on the internal linear SVM-based classification scores, thus making classification significantly more accurate. The RFE feature selector exhibited 85.29% accuracy in binary classification settings. However, in the multiclass classification setting, we obtained 60.78% accuracy.

We utilized the H-ELM framework as the main classification algorithm. Moreover, our noteworthy results and approaches for multiclass classification could assist with the establishment of a clinical diagnosis.

### Comparison with Previous Studies

Many studies have been conducted to classify ADHD subjects. Studies based on the use of a binary setting reported accuracies of 77% [42], 65% [43], and 65% [44]. Specifically, a study by [19], for which an accuracy of 90.18% was reported, was conducted only on subjects from the Peking University contribution to the ADHD-200 dataset for binary classification. In our study we used H-ELM, which is the most advanced classification framework for both binary and multiclass classification and has not been used by any other neuroimaging study to date. This framework has the benefit of an embedded ELM-based sparse feature autoencoder. Although the performance of the basic ELM classifier was found to be superior to that of the SVMs, it exhibits lower performance than H-ELM in every setting for both binary and multiclass classification. On the other hand, in a recent study by [45], a classification score of 95% by PCA-based feature optimization and a fully connected cascade artificial neural network classifier for binary settings was claimed. Furthermore, [46] reported a classification score of 82.7% in the binary case and 69.2% in multiclass settings with SVM by using the resting state functional connectivity maps of fMRI data. In addition, one study performed multiclass classification on ADHD, autistic, and normal children with an accuracy rate of 68.2% [47]. Another study by [22] reported 56.87% accuracy in multiclass settings by using single features and 57.71% by using multimodal features and it was ranked 6^th^ in the ADHD-200 competition. However, our results showed higher performance than any study on ADHD in multiclass classification settings by using only structural data, to the best of our knowledge. We used a balanced design approach to select the number of subjects (53 from each diagnosis group) from the ADHD-200 dataset. Among the aforementioned studies, there is no other similar experimental setting for multiclass classification of MRI cortical data; therefore, we cannot directly compare the accuracy of our results to those obtained in any previous research.

### Clinical Significance of ANOVA Results

The significant regions from our ANOVA result play an important role to obtain highly accurate differential diagnosis when used in combination with the other significant features that did not form part of the outcome of the ANOVA analysis. The following description of the regions obtained from our ANOVA analysis may have clinical significance:

In most neuroimaging studies regarding ADHD, the frontal-striato-parietal circuitry and default mode network has been suggested to be involved in ADHD [48]. Especially, the lateral fronto-striato-parietal circuit relates to attention/cognition [49], and the prefrontal cortex relates to working memory [50], executive function [51], motor control [52], and inhibitory control [53]. In addition, reward/motivation were shown to be related to the ventral striatum and ortbitofrontal cortex [54]. Moreover, the central sulcus was also reported to be associated with ADHD [18]. These regions showed significant differences between the control and ADHD groups. In our ANOVA result, multiple areas of the frontal cortex, including the MFG, precentral gyrus, and postcentral gyrus in the parietal cortex showed significantly higher cortical thickness in the TDC group. Thus, our findings are consistent with these results, and this might contribute to the high classification accuracy of our model. On the other hand, although not many reports were published, in the temporal lobe, it is evident from [55] that the cortical thickness differs significantly in ADHD patients as compared to the normal control group. The middle temporal gyrus is associated with language abilities, visual perception, multimodal sensory integration, and semantic memory processing. Our findings are consistent with these results.

### Significant Cortical Features for ADHD Multiclass Classification

Many previous studies mention that, even if we may find some specific regions with a high order of significance through group differences, still, the features extracted from those regions may not be able to yield high classification accuracies [56]. Group analysis-based features selection does not take into account those regions that have an area smaller than the group analysis output cluster size at a certain threshold value of the significance level. There exists a strong possibility that the regions discarded during group analysis may contain significant information that can be used to discriminate the diagnosis groups. However, the significant features found in our study for classification overlap with those regions that are found to be significant in terms of group differences through the ANOVA test.

In our ANOVA and post-hoc tests, we found that the TDC group showed a higher cortical thickness than the other two groups overall. Moreover, most regions we identified were included in the frontal lobe. Therefore, the most important regions for classification purposes were included in the frontal lobe. A previous study [4] reported that ADHD children showed delayed cortical maturation at an average of 3 years later than that of normal children [48]. Furthermore, most ADHD studies consistently demonstrated the finding that prefrontal and precentral areas were reduced in ADHD patients [57]. Our findings agree with these previous results. Table 6 presents all the significant features for multiclass classification. Indices of the cortical features are based on the DKT31 labeling protocol [24], as provided in Table 3.

**Table 6:** Most Significant Features for Classification.

Feature Measure (*fM*)	Left Hemisphere (index)	Right Hemisphere (index)
Mean Cortical thickness (1 - 64)	All (1 - 32)	All (1 - 32)
Surface Area (65 - 128)	26 and 32	26 and 32
Folding Indices (129 - 192)	None	None
Mean Curvature Indices (193 - 256)	None	None
Volume (257 - 320)	All except 4, 30, and 32	All except 4, 30, and 32

Second, during adolescence, normal children experience a cortical thinning phase [58]. Consistent with these findings, the top cortical features in our results can be used as promising biomarkers for automated ADHD classification. We found the surface area of the superior frontal lobe and mean surface area of the whole cortex to be the major contributors for the differential diagnosis of ADHD along with the cortical thickness and volume of the whole cortex by using multiclass classification. The folding indices and the curvature indices were found to be the most insignificant features.

In addition to the multiclass features reported in this paper, one important finding of the current study is that the significant cortical features are dataset dependent therefore, we cannot label any cortical feature as a global biomarker for ADHD classification. Our binary classification results indicate that the significant features are subject to change when we change the subgroup of ADHD for feature optimization. Therefore, it is presently very difficult to claim that any of the cortical features would be appropriate global biomarkers for ADHD discrimination.

### Limitations

The major limitation of this study was the imbalanced number of subjects from each diagnosis group, shared from each site. Site bias was intrinsically present in the dataset and it was treated as default. IQ data were missing for some of the subjects; therefore, we did not use IQ information in our study. We only selected the 53 subjects that were statistically matched from each diagnosis sub-group to enable us to conduct a balanced design study. To date, there is no clear guideline available as to the significant anatomical regions that can be used for the differentiation of ADHD subgroups by machine-learning methods; thus, we had to use grid search methodology to identify the most significant anatomical regions for the differential classification of ADHD.

### Conclusion

In conclusion, RFE feature selection was applied to ELM, SVM, and H-ELM classifiers, of which H-ELM achieved higher accuracy for both binary and multiclass classification with ADHD neuroimaging data. In addition, our noteworthy multiclass result could assist with clinical diagnosis of ADHD for subtype separation. Moreover, the top cortical features were consistent with previous neuroimaging studies involving ADHD subjects. This kind of multiclass classification approach is challenging; however, we hope that our study will inspire further studies with other clinical data. In a future study, we will perform a multimodal classification with only ANOVA-based features acquired from structural and functional brain MRI data to assess whether they can serve as a good predictor of the test data and increase the classification performance.
